# Overexpression of Endogenous Retroviruses and Malignancy Markers in Neuroblastoma Cell Lines by Medium-Induced Microenvironmental Changes

**DOI:** 10.3389/fonc.2021.637522

**Published:** 2021-05-07

**Authors:** Lisa Wieland, Kristina Engel, Ines Volkmer, Anna Krüger, Guido Posern, Malte E. Kornhuber, Martin S. Staege, Alexander Emmer

**Affiliations:** ^1^ Department of Neurology, Medical Faculty, Martin Luther University Halle-Wittenberg, Halle, Germany; ^2^ Department of Surgical and Conservative Pediatrics and Adolescent Medicine, Medical Faculty, Martin Luther University Halle-Wittenberg, Halle, Germany; ^3^ Institute for Physiological Chemistry, Medical Faculty, Martin Luther University Halle-Wittenberg, Halle, Germany

**Keywords:** endogenous retrovirus, human endogenous retrovirus, neuroblastoma, CD133, CD200, biomarker

## Abstract

Neuroblastoma (NB) is the commonest solid tumor outside the central nervous system in infancy and childhood with a unique biological heterogeneity. In patients with advanced, metastasizing neuroblastoma, treatment failure and poor prognosis is often marked by resistance to chemo- or immunotherapy. Thus, identification of robust biomarkers seems essential for understanding tumor progression and developing effective therapy. Here, we have studied the expression of human endogenous retroviruses (HERV) as potential targets in NB cell lines during stem-cell medium-induced microenvironmental change. Quantitative PCR revealed that relative expression of the HERV-K family and HERV-W1 *ENV* were increased in all three NB cell lines after incubation in stem-cell medium. Virus transcriptome analyses revealed the transcriptional activation of three endogenous retrovirus elements: HERV-R *ENV* (ERV3-1), HERV-E1 and HERV-Fc2 *ENV* (ERVFC1-1). Known malignancy markers in NB, e.g. proto-oncogenic MYC or MYCN were expressed highly heterogeneously in the three investigated NB cell lines with up-regulation of MYC and MYCN upon medium-induced microenvironmental change. In addition, SiMa cells exclusively showed a phenotype switching from loosely-adherent monolayers to low proliferating grape-like cellular aggregates, which was accompanied by an enhanced CD133 expression. Interestingly, the overexpression of HERV was associated with a significant elevation of immune checkpoint molecule CD200 in both quantitative PCR and RNA-seq analysis suggesting tumor escape mechanism in NB cell lines after incubation in serum-free stem cell medium.

## Introduction

In the course of evolution, a large number of retroviral elements have entered the genome of vertebrates. Since integration occurred by infection of the germ line with their exogenous relatives, the resulting proviruses of the so-called endogenous retroviruses (ERV) can be transmitted vertically as a host allele (1, 2). The human genome is comprised of approximately 8% of such elements (3, 4). During their long persistence in the genome, the proviruses suffered from multiple inactivation or silencing mechanisms that lead to disruption of open reading frames (ORF) by mutations and to defective protein products in nearly all human HERV (5). Nevertheless, there are few HERV with almost complete ORF, which are able to form functional proteins even if their intracellular trafficking seems to be inefficient (6).

As most controversially discussed HERV, the envelope (*ENV*) of the HERV-W locus on chromosome 7 (NCBI accession no.: NP_001124397.1; also known as ERVWE-1) can be mentioned. The encoded protein called syncytin-1 is expressed in the placenta, where it mediates cytotrophoblast fusion to the syncytiotrophoblast layer based on its fusogenic properties (7, 8). On the other hand, activation of HERV-W ENV has been associated with neurological disorders like multiple sclerosis (MS) due to its localization in brain lesions and detection of anti-HERV-W antibodies *in sera* of MS patients (9–11). In addition to the usual increase in autoreactive antibodies in the course of activation of the immune system (12), abnormally expressed HERV-W ENV has been shown to trigger inflammatory cascades including polyclonal activation of T lymphocytes [reviewed in (13)]. Another HERV that was associated more recently with autoimmune disorders is the ENV of HERV-Fc1 (NCBI accession no.: XM_011531085.2) (14, 15). Interestingly, the HERV-Fc family has an only limited expansion with six known proviruses in the human genome (16). Among the HERV families, HERV-K (HML-2) is the most active family, which comprises several complete members due to still ongoing fixation in the human population (17–19). Stronger expression of HERV-K family members including their group-specific antigens (GAG) has been mainly identified in tissues and cell lines established from different types of tumors including germ cell tumors, lymphoma, sarcoma and melanoma (20–25). Like melanoma, neuroblastoma (NB) is a tumor originating from cells of the neural crest and unique for its heterogeneity. NB is the most frequent solid tumor outside the central nervous system in infants and children with an incidence of approximately 10 cases per million children under 15 years of age (26). An important prognostic marker is MYCN amplification, which is associated with deregulated growth and proliferation and can be found in 30–40% of high risk NB (27). MYCN is a member of the MYC family of transcription factors. Another family member, c-myc (MYC), shares oncogenic ability to sustain multiple pathways leading to malignancy, but is reported to be expressed only in a small group of advanced NB showing poor clinical outcome identical to that of patients with amplification of the *MYCN* gene (28, 29). Although overall outcome of patients have been improved in the last decades, survival for patients with high risk NB (stages 3 and 4 by the International Neuroblastoma Stating System INSS) remains poor due to chemotherapy failure or unresponsiveness to checkpoint blockade immunotherapy (30–32). In the course of focusing on individualized targeted therapy for more effective treatment, modulation of tumor microenvironment seems to be crucial (33, 34). In this context, lack of immunotherapeutic targets, like programmed cell death ligand-1 (PD-L1), in CD24 overexpressing NB, as well as amplification of multidrug resistance-associated genes and overexpression of immune checkpoint molecule CD200 in NB is of particular interest (35–38).

Studies from an Italian group strongly suggest that the stem cell-like CD133 positive subtype of melanoma cells is promoted by HERV-K activation in response to microenvironmental change (39). It remains unknown, whether such effects also occur in other neural crest-derived tumor cells. Therefore, we investigated the expression of above mentioned NB malignancy markers and selected HERV in three NB cell lines in standard and stem cell-promoting media with or without serum by quantitative real time PCR (RT-qPCR). For the RT-qPCR analysis, we focused on the *ENV* of human HERV-W1 and HERV-Fc1 due to their strong association with neurological disorders. In addition, the *GAG* region (HERV-K *GAG*) of the HERV-K (HML-2) family was investigated, because of its putative role in the progression of several tumor malignancies (19). In accordance, we previously reported a robust HERV-K *GAG* expression in soft tissue sarcoma patients, which was significantly correlated with clinicopathological features, such as shortened relapse-free survival, and hypoxia-related gene expression (24). In addition, the targeting of HERV-K *GAG* is beneficial as it allows the detection of numerous members of the most biologically active HERV-K (HML-2) family, which might be hampered by the presence of intact (type 1) and non-intact ORF (type 2) in the ENV region (18). Considering the broadest distribution across HERV families and the overall high sequence similarity, the detection of additional HERV-K families (e.g. HML-6) is not excluded by our study. In addition, we analyzed activation of retroviral sequences by mapping RNA-seq reads against a synthetic virus metagenome.

## Materials and Methods

### Cell Lines

In this study the human NB cell lines SH-SY5Y (40), IMR-32 (41) and SiMa (42) were used (all from the German Collection of Microorganisms and Cell Cultures GmbH, Braunschweig, Germany). All cell lines were cultured as adherent or loosely-adherent (SiMa) cells in DMEM medium supplemented with 10% (v/v) heat-inactivated fetal bovine serum (FBS) and penicillin-streptomycin at 37°C in a humidified 5% CO_2_ atmosphere (all reagents by Life Technologies, Carlsbad, CA, USA). Twice weekly the cells were passaged after detachment with 0.05% trypsin/EDTA solution (Life Technologies, Carlsbad, CA, USA) for 30–60 s at room temperature and were seed out at 3x10^6^ cells per 75 cm^2^ flask (SH-SY5Y cells and IMR-32 cells) or at 5 × 10^6^ cells per 75 cm^2^ flask (SiMa cells), respectively.

To investigate the medium-induced microenvironmental changes, the three NB cell lines were seed out at 1 × 10^6^ cells (SH-SY5Y cells and IMR-32 cells) or at 3 × 10^6^ cells (SiMa cells) per 25 cm^2^ flask and cultured either in DMEM complete medium, stem-cell medium Panserin 401 (PAN-Biotech GmbH, Aidenbach, Germany) supplemented with 10% FBS and penicillin–streptomycin or Panserin 401 medium with penicillin–streptomycin for 72 h at 37 °C in a humidified 5% CO_2_ atmosphere. Morphological analyses were performed by phase contrast microscopy using Keyence microscope BZ-X810 and BZ-X800 Analyzer software version 1.1.1.8 (Keyence, Itasca, IL, USA).

### RNA Extraction, cDNA Generation and Quantitative Real-Time PCR

Cells were harvested after 72 h and total cellular RNA was isolated using NucleoSpin RNA kit (Machery-Nagel GmbH & Co. KG, Düren, Germany) following the manufacturer’s instructions. Transcription into cDNA was performed using 1 μg total RNA in 16 μl nuclease-free water and 4 μl qScript cDNA 5× SuperMix (QuantaBio, Beverly MA, USA). The mix was incubated for 5 min at 25°C, followed by 30 min at 42°C and finally for 5 min at 82°C.

For quantitative real-time PCR (RT-qPCR) each reaction contained 0.5 µl of cDNA, 500 nM of forward and reverse primer, 5 µl PowerUP SYBR Green 2× Master Mix (Applied Biosystems by Thermo Fisher Scientific, Waltham MA, USA) and 4 µl of nuclease-free water. All used primers were purchased from Invitrogen Thermo Fisher Scientific (Waltham, MA, USA) and were listed in **Table 1**. The amplification protocol included an initial denaturation step at 95°C for 10 min, followed by 40 cycles with denaturation at 95°C for 15 s and primer annealing, amplification and extension at 60°C for 60 s. Two technical replicates were measured for each sample in three independent experiments (biological replicates). The analysis was performed using QuantStudio3 and QuantStudio Design and Analysis Software v.1.4.3 (Thermo Fisher Scientific). The quantification of relative mRNA levels was performed using the 2^−ΔΔCt^ method (44). Hypoxanthine phosphoribosyltransferase 1 (HPRT1) was used as reference gene for normalization and the median of all samples was set as 1.

**Table 1:** Primers used for quantitative real time PCR.

Target	Exemplary accession number (reference)	Sequence of forward (f) and reverse (r) primer (5’-3’)
**ABCC5**	NM_001023587	f: CGAAGGGTTGTGTGGATCTT
		r: TCTCCCCTCCCTCAGATTTTT
**CD24**	NM_013230	f: ACCCACGCAGATTTATTCCA
		r: ACCACGAAGAGACTGGCTGT
**CD133**	NM_006017	f: GCCACCGCTCTAGATACTGC
		r: TGTTGTGATGGGCTTGTCAT
**CD200**	NM_005944	f: AAGTGGTGACCCAGGATGAAA
		r: AGGTGATGGTTGAGTTTTGGAG
**HERV-Fc1 ENV**	XM_011531085	f: CTCCCCATCTCTCTGGTGC
		r: TGAGGAGGCTGGTTTCTACTAAG
**HERV-K GAG**	JN675025 (23)	f: GGCCATCAGAGTCTAAACCACG
		r: CTGACTTTCTGGGGGTGGCCG
**HERV-W1 ENV**	NM_014590 (43)	f: TGCTAACCGCTGAAAGAGGG
		r: CGAAGCTCCTCTGCTCTACG
**HPRT1**	NM_000194	f: ACCAGTCAACAGGGGACATAA
		r: CTTCGTGGGGTCCTTTTCACC
**MYC**	NM_002467	f: GGCTCCTGGCAAAAGGTCA
		r: CTGCGTAGTTGTGCTGATGT
**MYCN**	NM_001293228	f: TGATCCTCAAACGATGCCTTC
		r: GGACGCCTCGCTCTTTATCT

### Statistics

For comparison of relative mRNA levels measured with RT-qPCR, two-way ANOVA followed by Tukey test was performed using GraphPad Prism (version 8.0.0 for Windows, GraphPad Software, San Diego, California USA). Statistical significance was indicated by asterisks (**, p <0.01; ***, p <0.001; ****, p <0.0001).

### RNA-seq

Generation of RNA-seq data was performed by Novogene UK Co., Ltd. (Cambridge, United Kingdom) using Illumina Novaseq6000 system. The quantification of human transcripts mapping to genome version GRCh38/hg38 was calculated as Fragments Per Kilobase per Million reads (FPKM) by Novogene. RNA-seq data can be downloaded from the NCBI Short Read Archive (SRA) under BioProject PRJNA684790. For quantification of ERV expression, reads were mapped against a synthetic virus metagenome, which consists of 119 individual human endogenous viral sequences including three endogenous bornavirus-like elements with almost complete ORF for their *GAG*, *POL* and *ENV* genes, four sequences of housekeeping genes and 124 sequences from unrelated exogenous viruses or non-human endogenous viruses used as spacers. The HERV-K (HML-2) family has more than 100 integrated copies in the human genome, of which we added the 92 full-length sequences to our virus metagenome. All sequences were collected from the nucleotide database from the National Center for Biotechnology Information (NCBI). For detailed information, refer to Engel et al. (45). The Galaxy server at usegalaxy.org (46) was used for mapping of the paired-end reads by Bowtie2 analysis and quantification of all uniquely mapped reads using FeatureCounts (47). To obtain the HERV family specific FPKM, the fragment read counts and the gene length of a family was calculated by summarization of individual family members.

### Flow Cytometry

Cells were harvested after 72 h under medium-induced microenvironmental changes. Therefore, loosely-adherent or suspensory cells were loosened by gentle pipetting. Adherent cells were harvested by detachment with 0.05% trypsin/EDTA solution. For antibody staining, cells were resuspended in ice-cold PBS with 2 mM EDTA and 5 μl of the antibody solution was added. Cells were stained using CD200-APC (Miltenyi Biotech, Bergisch Gladbach, Germany) or IgG1-APC control (BD Biosciences, Franklin Lakes, NJ, USA) for 30 min at 4°C in the dark. Unbound antibody was removed with 1 ml PBS/EDTA solution and centrifugation for 10 min at 300 ×*g*. Finally, cells were suspended in 0.5 ml PBS/EDTA solution and analyzed on a LSRII cytometer using the FACSDiva software version 8.0.1 (BD Biosciences, Franklin Lakes, NJ, USA).

## Results

### Trancriptional Activation of Tumor Progression Markers and HERV by Medium-Induced Microenvironmental Changes in Neuroblastoma Cell Lines

**Figure 1:**
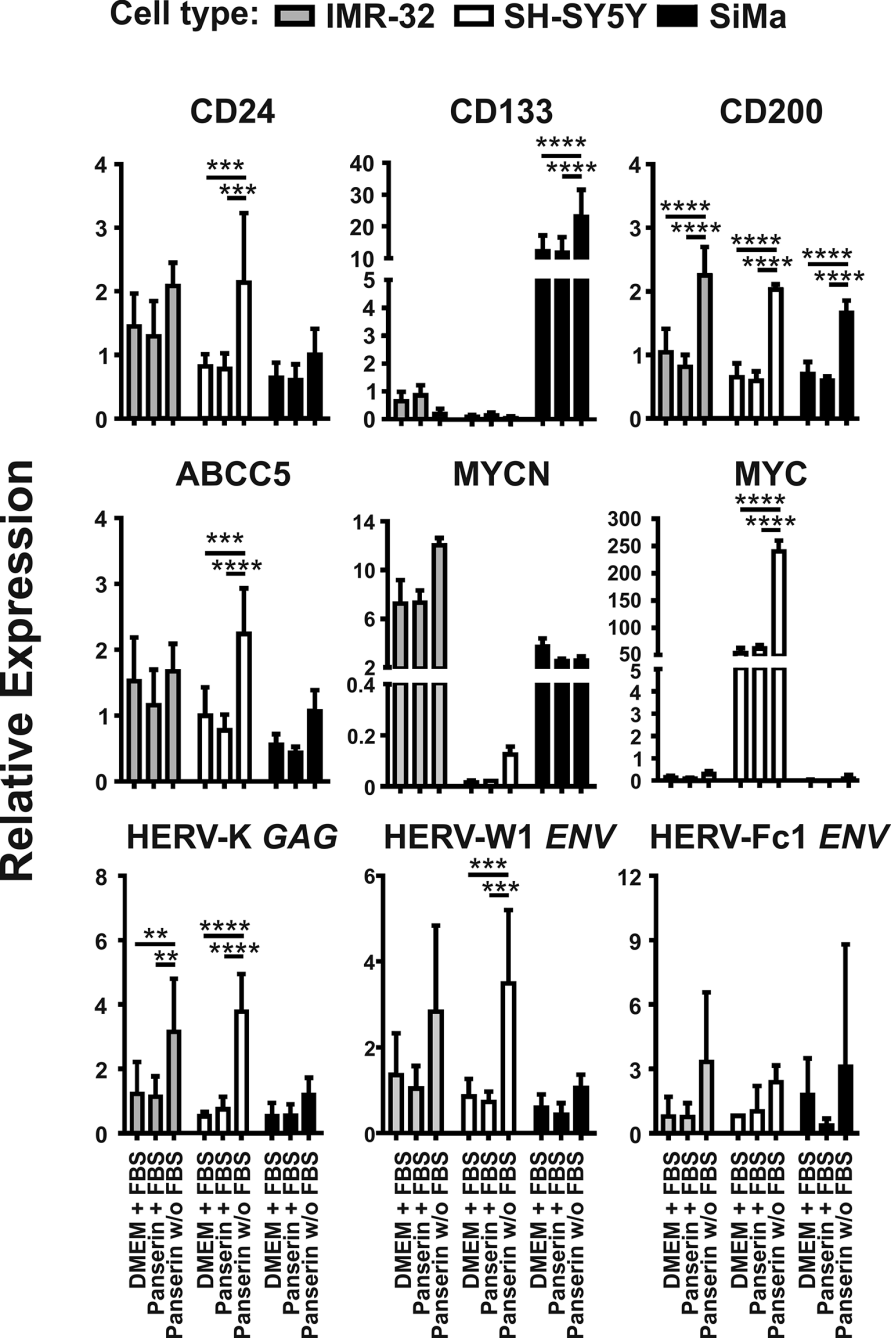
Promotion of malignancy markers by medium-induced microenvironmental changes is accompanied by HERV induction in NB cell lines. Expression of proposed malignancy markers in NB and three selected HERV transcripts was assessed by RT-qPCR. Relative expression of the indicated genes was represented by 2^−ΔΔCt^ with normalization to HPRT1 and the overall median. The graphs show mean values and standard deviations of three individual experiments (biological replicates) determined in duplicates (technical replicates). Statistics: two-way ANOVA with Tukey test; ****p < 0.0001; ***p < 0.001; **p < 0.01.

In order to investigate the expression of selected HERV and cellular markers involved in NB tumor progression or invasiveness under microenvironmental modifications, the three NB cell lines SH-SY5Y, IMR-32 and SiMa were cultured in serum-supplemented DMEM standard medium or serum-supplemented stem cell medium (Panserin 401), or Panserin without serum. Using RT-qPCR analyses, the transcript levels of all investigated HERV were shown to be up-regulated upon exposure to serum-free stem cell medium (**Figure 1**). Both, elevation of HERV-K *GAG* in SH-SY5Y cells (p < 0.0001) and IMR-32 cells (p < 0.01), as well as activation of HERV-W1 *ENV* in SH-SY5Y cells (p < 0.001) were statistically significant. In SiMa cells, a tendency for increase of the relative HERV expression was observed (black bars). No significant regulation was observed for HERV-Fc1 *ENV*, while the expression was comparatively low with CT values around 30. Concordantly, enhanced expressions of CD24 and CD200 were observed in all three NB lines following cultivation in stem cell media, which was highly significant (p < 0.0001) for CD200 compared to both serum-supplemented incubations. An increased CD200 expression at protein level was confirmed in all three NB cell lines by flow cytometry analyses (**Supplementary Figure 1**). Furthermore, relative expression of ABCC5, also known as multidrug resistance-associated protein 5, was increased in SiMa cells and significantly increased in SH-SY5Y cells, whereas no effect was observed in IMR-32 cells. The members of the proto-oncogene MYC family, MYCN and MYC, were expressed heterogeneously in the three studied NB lines. High expression of MYCN was seen in SiMa cells and IMR-32 cells, but not in SH-SY5Y cells. Upon medium-induced microenvironmental changes, MYCN levels were increased by factor 1.6 in IMR-32 cells only. In contrast, MYC expression was exclusive for SH-SY5Y cells and increased by at least factor 4 (p < 0.0001) in serum-free stem cell medium. Interestingly, SiMa was the only NB cell line that underwent morphological changes from loosely-adherent monolayers to low proliferating grape-like cellular aggregates upon exposure to serum-free stem cell medium (**Supplementary Figure 2**), accompanied by highly significant (p < 0.0001) enrichment of CD133 expression. The NB cell lines SH-SY5Y and IMR-32 did show neither phenotype switching nor transcriptional activation of CD133 during medium-induced microenvironmental changes.

### Differential Gene Expression Pattern in HERV-Expressing Neuroblastoma Cell Lines

**Figure 2:**
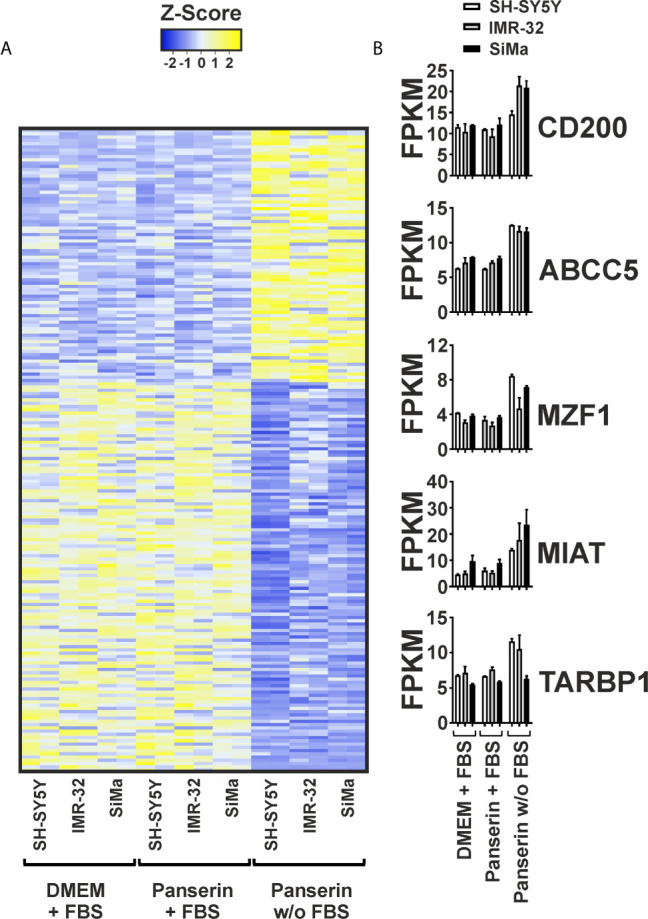
Differential gene expression pattern induced by serum-free medium in three NB cell lines. Differential gene expression of three NB cell lines cultured in DMEM or Panserin with FBS or serum-free Panserin was assessed by RNA-Seq. Data from two individual experiments (biological replicates) are presented. **(A)** Heatmap of all genes showing a high correlation with RT-qPCR-based expression of HERV-K *GAG* (Pearson coefficient: ≥0.7 or ≤−0.7), as well as a differential expression with fold-change ≥1.4 in medium with or without FBS, respectively. The difference of the FPKM above (yellow) and below (blue) the mean value in relation to the standard deviation is shown as Z-score. Heatmap was generated using www.heatmapper.ca. **(B)** The graphs show mean values with standard deviation of selected transcripts from the gene panel described in **(A)**.

Secondly, we investigated the overall gene expression in medium-induced HERV-transcriptional active NB cell lines. Therefore, RNA-seq data were collected in duplicates from all three NB cell lines in serum-supplemented standard and stem cell media or stem cell medium without serum. After mapping against the human genome GRCh38/hg38, the 2^−ΔΔCT^-values were used to filter for transcripts that showed either a high positive (r ≥0.7) or negative (r ≤−0.7) correlation to expression of HERV-K *GAG*. The strongest up- and downregulated genes are presented in **Figure 2**. The list of all 198 up- and downregulated genes can be found in the supplement of this manuscript (**Supplementary Tables 1, 2**). **Figure 2A** shows that stem cell medium led to a shift in gene expression of all three NB cell lines, accompanied by the formation of individual expression signatures. No differences in the overall expression pattern were observed between the serum-supplemented standard and stem cell media incubated cells, indicating serum deprivation as the major factor affecting transcription. This, together with increasing levels of CD200 and ABCC5 in serum-free medium, shows that the RNA-seq analysis is in line with our previously observed RT-qPCR results (**Figure 2B**). Furthermore, TAR (HIV-1) RNA Binding Protein 1 (TARBP1) was increased by 1.46 times in NB incubated in serum-free stem cell medium, which might be of interest regarding potential host interaction partners of the activated HERV. As two of the 78 strongest up-regulated genes, the long non-coding RNA (lncRNA) Myocardial Infarction Associated Transcript (MIAT) by fold change of 2.11 and the transcription factor Myeloid Zinc Finger 1 (MZF1) by fold change of 1.87 were observed (**Figure 2B** and **Supplementary Table 2**). In addition, enhanced expression of the P21 Activated Kinase (PAK) 3 (fold change: 1.51) and PAK5 (fold change: 1.79) were observed. In contrast, 120 genes were found to be down-regulated by serum-free medium and correlated negatively with HERV expression (**Figure 2A** and **Supplementary Table 2**). Hereby, Inhibitor of DNA Binding Protein (ID) 1 and ID2 that are typically expressed by cells of the neural crest were both among the strongest decreased genes. Furthermore, vimentin (VIM), a known regulator of tumor suppressor p21, was reduced in all three NB cell lines after exposure to serum-free media.

### Identification of Additional Stem Cell Medium-Induced HERV Members by Using a Virus Metagenome

**Figure 3:**
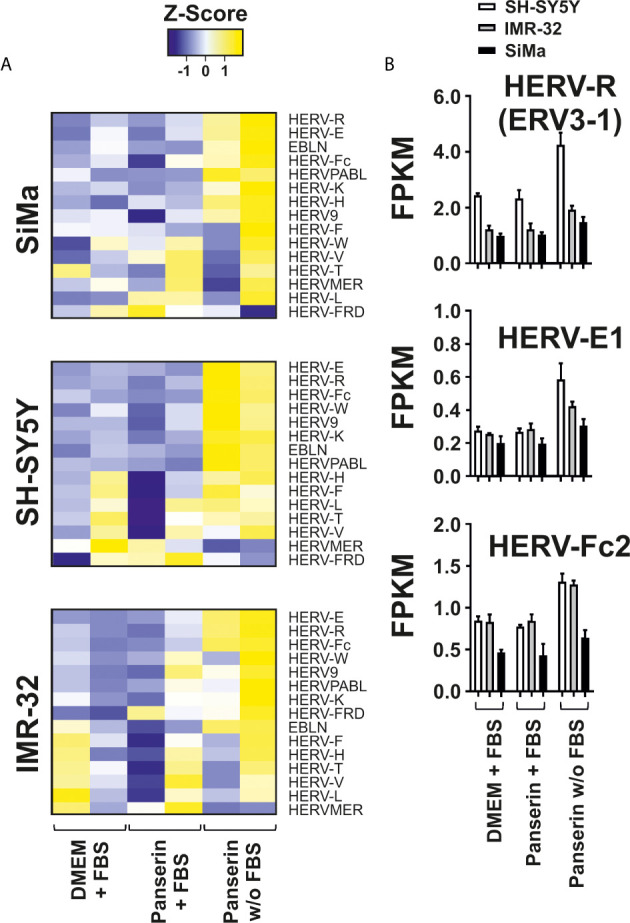
Differential expression of HERV families induced by serum-free medium in three NB cell lines. Analysis of HERV and bornavirus-like element transcription in three NB cell lines cultured in DMEM or Panserin with FBS or serum-free Panserin medium was assessed by RNA-seq of two individual experiments (biological replicates). **(A)** Heatmaps of HERV family expression in each cell line. FPKM of virus families were calculated with respect to the sum of fragment counts and gene lengths of all family members. The families were ranked from highest to lowest differential expression in serum free medium. The difference of the sum of FPKM above (yellow) and below (blue) the mean value in relation to the standard deviation is shown as Z-score. Heatmap was generated using www.heatmapper.ca. **(B)** The graphs show mean values with standard deviation of FPKM from selected members of the three strongest up-regulated HERV families in all NB cell lines.

To study viral transcription and their potential activation in a pathogenic context using RNA-seq, we designed a combined virus metagenome including a collection of exogenous viruses, endogenous retrovirus elements and other endogenous viral elements (EVE), e.g. endogenous bornavirus sequences. Using this virus metagenome, we were able to investigate the differential expression pattern of 115 HERV elements representing 14 HERV families and three EVE of the bornavirus family (EBLN-1, EBLN-2 and EBLN-3P) in the three NB cell lines under medium-induced microenvironmental change. We found that overall expression of EVE was low. But half of studied endogenous virus families were up-regulated upon removal of serum supplementation in stem cell medium (**Figure 3A**). Interestingly, three HERV elements showed strongest differential expression across all three NB cell lines: the *ENV* of HERV-R (ERV3-1; NCBI accession no.: NC_000007.14:64990356-65006687), the full-length HERV-E1 (NCBI accession no.: AB062274.1) and the *ENV* of HERV-Fc2 (NCBI accession no.: AC073236.8:162447-165176). As shown in **Figure 3B**, HERV-E1 expression was most increased by factor 2.1 in SH-SY5Y cells and by factor 1.6 in IMR-32 cells. In SiMa cells, ERV3-1 was strongest upregulated with a fold change of 1.4 and here the highest expression of the bornavirus like-family was observed, too.

## Discussion

In the present study, we have investigated the expression of HERV as potential targets in NB cell lines during stem-cell medium-induced microenvironmental change by RT-qPCR and RNA-seq. For RT-qPCR analyses we initially focused on HERV-K due to its well-described relationship in a number of cancers including neural crest-derived tumors such as melanoma (22, 39, 48–50). Our results suggest a strong correlation of HERV-K *GAG* with stressful cell culture conditions by serum-depleted stem cell medium in all three NB cell lines (**Figure 1**). In this context, studies on human pluripotent stem cells (PSCs) suggest HERV-K (HML-2) activation in early stem cell development (51, 52) and that the differentiation of PSCs into neuronal cells is promoted by silencing of its *ENV* (53). It is of particular interest, that Wang’s group were not able to demonstrate similar results with GAG proteins. This might be a result of an only moderate HERV-K *GAG* activation in PSC-like cells, as we observed for SiMa cells after stem cell media induction. Instead, elevation was significant in SH-SY5Y cells (p < 0.0001) and IMR-32 cells (p < 0.01) where no morphological changes were observed (**Figure 1** and **Supplementary Figure 2**).

Besides up-regulation of HERV-K *GAG*, enhanced expression of HERV-W1 *ENV* with significance in SH-SY5Y cells (p <0.001) and to a lesser extend up-regulation of HERV-Fc1 *ENV* were observed as consequence of stem cell medium induction (**Figure 1**). In addition, virus transcriptome analyses revealed activation of three endogenous retrovirus elements: HERV-R (ERV3-1), HERV-E1 and HERV-Fc2 (**Figure 3**). HERV-R transcripts have broad expression in normal tissue and due to its overexpression in the first trimester of pregnancy an immunosuppressive function in the mother-fetus interaction was suggested (54, 55). ERV3-1 is the only copy of approximately 40 ERV3-like elements in the human genome that has a complete ORF for its ENV (56). Interestingly, the expression of this full-length ENV is associated with several tumor entities including colorectal (57), ovarian (58) and endometrial carcinoma. Of interest is that during endometrial carcinoma progression, ERV3-1 *ENV* is co-expressed with six other *ENV* (HERV-W1, HERV-T, HERV-Fc2, HERV-H1-3, HERV-V1, HERV-E1) and showed significantly increased expression in more undifferentiated grade 3 tumors compared to differentiated grade 1 tumors (59). In accordance with the results from endometrial carcinoma, elevated expression of HERV-E1, HERV-R, and also HERV-K were observed in ovarian cancer (58). Several reports suggest a putative pathogenic role in systemic lupus erythematosus as expression of provirus elements was found to be enhanced compared to healthy individuals and to correlate with disease progression (60–63). Altogether, this demonstrates the genetic variety of HERVs in the pathogenesis of autoimmune disorders and malignancies, even in the limited subset of NB cell lines shown here. HERV transcriptome analysis of tumor biopsy samples from NB patients might be helpful for identification of putative HERV targets that can be used for development of anti-HERV antibodies. Such antibodies might be tools for a more personalized and, at best, more effective therapy, as it is already established for *HER2* in breast cancer patients (64). The stem cell medium-induced element identified within our virus metagenome analysis, HERV-Fc2 *ENV* with location on chromosome 7q36 has not been investigated extensively in the past. Due to its activation in all three NB cell lines upon tumor microenvironmental change, we propose HERV-Fc2 *ENV* as a putative target in NB that should be further studied.

In RT-qPCR analyses, IMR-32 cells and SH-SY5Y cells had either a distinct MYCN (IMR-32 cells) or MYC (SH-SY5Y cells) positive phenotype, which has been pronounced under stem cell incubation significantly in SH-SY5Y cells (p < 0.0001). Another fact exemplifying the heterogeneity of NB was the CD133 positive character of the SiMa cell line and its medium-induced morphological change from loosely adherent monolayers to low proliferating grape-like cellular aggregates. Phenotype switching in SiMa cells was further accompanied by significant increase of CD133 levels assessed by RT-qPCR (p < 0.0001) and by RNA-seq with a fold change of 1.56. Altogether, this indicates that SiMa cells used in our study are so-called I-type NB (65). I-type cells were shown to be significantly more malignant than N- or S-type NB independent of MYCN amplification and suggested as cancer stem cell population according to their CD133 positive background (66, 67). Besides these differences, expression of CD24, CD200 and ABCC5 were upregulated upon stem cell-promoting conditions with highly significant fold changes for CD200 (p < 0.0001) in all three NB cell lines and for CD24 (p < 0.001) and ABCC5 (p < 0.001) in SH-SY5Y cells (**Figure 1**). Since overexpression of CD200 was reported in a variety of human tumors including multiple myeloma (68), neuroendocrine tumors (69), melanoma (70, 71), ovarian cancer (72), and very recently also in more than 90 % of NB samples (38), high correlation of CD200 and HERV-K *GAG* for the studied NB cell lines (r = 0.92) might be of special interest (**Figure 2**). This raises the question, if CD200 is activated by HERV expression upon microenvironmental change or *vice versa* considering possible signaling pathways. In the case of an initial HERV overexpression by e.g. exogenous viruses (73–75) HERV proteins from almost complete ORF might induce clonal deletion of lymphocytes in a T cell receptor V-beta specific manner resembling superantigens (13, 76). The polyclonal expansion of T lymphocytes that has been previously shown for a HERV-W protein *in-vitro* (77), lead to secretion of cytokines and consequently upregulation of CD200 by activated T cells (34). Of interest, CD4 positive and CD8 positive T cells of CD200^high^ NB were shown to produce less interferon gamma and tumor necrosis factor alpha thereby inhibiting anti-tumor immunity (38). Nevertheless, controversial results have been reported according the significance and role of CD200 expression in cancer progression indicating a certain dependence on the tumor type (69, 78, 79). The three NB cell lines used in this study showed an increased CD200 expression upon medium-induced microenvironmental change, both at the RNA level and at the surface protein level (**Supplementary Figure 1**). Though CD24 was not included in the list of most differentially expressed genes (**Figure 2** and **Supplementary Table 1**) due to a fold change smaller than 1.4, transcriptome analysis confirms enhanced expression in Panserin medium. Previous studies reported CD24 as an inhibitor for neurite outgrowth in mice and that expression was related to the differentiation state in human NB suggesting an activation of CD24 in less differentiated tumor samples (80, 81). This might be of interest, since morphological changes with loss of dendritic branching was solely observed in SiMa cells after 72 h of serum-free media incubation (**Supplementary Figure 2**), but may indicate also ongoing genetic reprogramming in SH-SY5Y cells and IMR-32 cells. In this context it is interesting that stem-cell medium induced down regulation of ID1 and ID2, as well as VIM, which are typically expressed by neural crest cells (82, 83) (**Figure 2** and **Supplementary Table 2**). An overexpression of ID1 was associated with several cancers and cancer-associated pathways (84). However, the reduced expression of the ID1 and ID2 might be indicative for impaired proliferation in serum-depleted media that we observed at least in SiMa cells.

Furthermore, we observed overexpression of MIAT under stem-cell promoting conditions in all studied NB cell lines (**Figure 2B** and **Supplementary Table 1**). This nuclear lncRNA (NCBI accession no.: NR_003491) is widely expressed in endothelial cells, Müller glia and neurons, but dysregulation of MIAT is associated with various heart diseases and nervous system tumors, as it is involved in the maintenance of proper microvascular and nervous function (85). Interestingly, silencing of MIAT was reported to correlate with down regulation of MYC leading to reduction of cell migration and promotion of basal apoptosis in the NB cell line SH-SY5Y (86). In our present study, the correlation of MYC and MIAT amplification seemed to be exclusive for SH-SY5Y cells, as expression of MYC was not detected in IMR-32 cells and SiMa cells (**Figure 1**). In contrast, differential expression analyses in stage 4 NB and stage 4S NB suggested that MIAT might be a “good survival lncRNA” which needs further investigation (87). Overexpression of transcription factor MZF1 detected by RNA-seq (**Figure 2** and **Supplementary Table 1**) was in line with previous reports of MZF1 association with poor clinical outcome in different tumor entities [reviewed in (88)] and especially NB tumor cell progression through modulation of tumor environment by facilitating aerobic glycolysis in NB cell lines (89). Last but not least, co-activation of TARBP-1 and HERV transcripts might be of special interest. Initially identified to bind with HIV type-1 transactivation response RNA to activate long terminal repeat (LTR) expression in the presence or absence of the viral transactivator Tat (90), it is reasonable to hypothesize that TARBPs might be able to activate nuclear LTR transcription of endogenous proviruses and consequently promote expression of HERV proteins if they possess intact ORF.

In summary, NB are very heterogeneous tumors hampering the identification of robust biomarkers. Our results strongly suggest enhancement of malignancy markers by medium-induced tumor microenvironmental change in RT-qPCR and RNA-seq, which is accompanied by activation of HERV transcription in all studied NB cell lines. To our knowledge, this is the first time that HERV-R *ENV* (ERV3-1), HERV-E1 and HERV-Fc2 *ENV* were reported to be associated with NB and should be investigated in further studies especially regarding their prevalence in more undifferentiated tumor cells. In addition, significant increase of immune checkpoint molecule CD200 indicating and possible activation of immune escape mechanisms, as well as TARBP-1 co-activation with HERV needs to be further explored. The expression analysis of HERV elements in patient specimens might lead to identification of new therapeutic targets, especially regarding the ongoing efforts in the production of HERV-targeting drugs.

## Data Availability Statement

The datasets presented in this study can be found in online repositories. The names of the repository/repositories and accession number(s) can be found below: NCBI Sequence Read Archive (SRA) (https://www.ncbi.nlm.nih.gov/sra/). Accession number: PRJNA684790.

## Author Contributions

Conceptualization: LW and MS. Methodology: LW, KE, and IV. Data curation: LW and MS. Visualization and original draft preparation: LW. Review and editing: LW, AK, GP, and MS. Supervision, resources, and project administration: AE, MK, and MS. All authors contributed to the article and approved the submitted version.

## Funding

The work was supported by grant ZS/2018/12/96228 (to University of Halle) from European Regional Development Fund within the local program “Sachsen-Anhalt WISSENSCHAFT Schwerpunkte”. We acknowledge the financial support of the Open Access Publication Fund of the Martin Luther University Halle-Wittenberg.

## Conflict of Interest

The authors declare that the research was conducted in the absence of any commercial or financial relationships that could be construed as a potential conflict of interest.
