# Supplementary material for: Overexpression of Endogenous Retroviruses and Malignancy Markers in Neuroblastoma Cell Lines by Medium-Induced Microenvironmental Changes
Source: Front Oncol. 2021 May 7;11:637522. doi: 10.3389/fonc.2021.637522 (PMC8138558; doi:10.3389/fonc.2021.637522)
Supplement: Supplementary file 1 [file DataSheet_1.pdf]

Supplementary Material for

Overexpression of Endogenous Retroviruses and Malignancy Markers in Neuroblastoma Cell Lines by Medium-Induced Microenvironmental Changes

Lisa Wieland, Kristina Engel, Ines Volkmer, Anna Krüger, Guido Posern, Malte E. Kornhuber, Martin S. Staege, Alexander Emmer

This file contains:

- **Supplementary Table STab.1 page 2**
- **Supplementary Table STab.2 page 4**
- **Supplementary Figure SFig. 1 page 7**
- **Supplementary Figure SFig. 2 page 8**

1 Supplementary Tables

Supplementary Table STab.1. Up-regulated genes expressed in three NB cell lines after culture in serum-free stem cell medium. Up-regulated genes were identified by RNA-Seq. Genes are ranked from highest to lowest fold change of FPKM in serum-free medium in the same order as shown in Figure 2 of the manuscript.

**Gene ID**	**Name**	***fold change***
ENSG00000277311	RF02247	3.331
ENSG00000274520	RF02246	2.602
ENSG00000152377	SPOCK1	2.354
ENSG00000184194	GPR173	2.271
ENSG00000250067	YJEFN3	2.265
ENSG00000225783	MIAT	2.106
ENSG00000185615	PDIA2	2.071
ENSG00000232931	LINC00342	2.023
ENSG00000245849	RAD51-AS1	1.969
ENSG00000197093	GAL3ST4	1.961
ENSG00000155980	KIF5A	1.920
ENSG00000264112	AC015813.1	1.883
ENSG00000114796	KLHL24	1.874
ENSG00000099326	MZF1	1.872
ENSG00000171004	HS6ST2	1.837
ENSG00000267595	AC060780.3	1.827
ENSG00000101349	PAK5	1.794
ENSG00000267523	AC008735.2	1.736
ENSG00000144362	PHOSPHO2	1.720
ENSG00000272240	AC004908.1	1.719
ENSG00000144834	TAGLN3	1.713
ENSG00000267002	AC060780.1	1.707
ENSG00000244879	GABPB1-AS1	1.695
ENSG00000151376	ME3	1.693
ENSG00000215252	GOLGA8B	1.671
ENSG00000091972	CD200	1.666
ENSG00000250467	AC105389.3	1.657
ENSG00000263272	AC004148.2	1.654
ENSG00000215397	SCRT2	1.654
ENSG00000267254	AC020928.1	1.651
ENSG00000018236	CNTN1	1.610
ENSG00000146966	DENND2A	1.602
ENSG00000108309	RUNDC3A	1.592
ENSG00000149809	TM7SF2	1.591
ENSG00000124406	ATP8A1	1.586

ENSG00000158292	GPR153	1.583
ENSG00000162004	CCDC78	1.581
ENSG00000133134	BEX2	1.576
ENSG00000114770	ABCC5	1.568
ENSG00000105270	CLIP3	1.561
ENSG00000135596	MICAL1	1.554
ENSG00000182901	RGS7	1.554
ENSG00000131067	GGT7	1.554
ENSG00000129003	VPS13C	1.551
ENSG00000169231	THBS3	1.537
ENSG00000104899	AMH	1.537
ENSG00000224032	EPB41L4A-AS1	1.521
ENSG00000145198	VWA5B2	1.517
ENSG00000077264	PAK3	1.515
ENSG00000160271	RALGDS	1.511
ENSG00000250959	GLUD1P3	1.495
ENSG00000272301	AP002360.3	1.494
ENSG00000175265	GOLGA8A	1.491
ENSG00000169733	RFNG	1.488
ENSG00000220205	VAMP2	1.481
ENSG00000034677	RNF19A	1.474
ENSG00000254064	AC105206.2	1.467
ENSG00000054793	ATP9A	1.467
ENSG00000059588	TARBP1	1.464
ENSG00000171533	MAP6	1.452
ENSG00000120324	PCDHB10	1.452
ENSG00000102078	SLC25A14	1.447
ENSG00000139636	LMBR1L	1.447
ENSG00000188785	ZNF548	1.439
ENSG00000196123	KIAA0895L	1.439
ENSG00000166924	NYAP1	1.433
ENSG00000105443	CYTH2	1.432
ENSG00000131584	ACAP3	1.432
ENSG00000167363	FN3K	1.430
ENSG00000177410	ZFAS1	1.425
ENSG00000100167	SEPT3	1.424
ENSG00000155093	PTPRN2	1.421
ENSG00000183098	GPC6	1.414
ENSG00000212694	LINC01089	1.412
ENSG00000234338	AC073349.2	1.407
ENSG00000258682	AL132989.1	1.406
ENSG00000162687	KCNT2	1.404
ENSG00000033627	ATP6V0A1	1.402

Supplementary Table STab.2. Down-regulated genes in NB cell lines after culture in serum-free medium. Down-regulated genes were identified by RNA-Seq. Genes are ranked from lowest to highest fold change of FPKM in serum-supplemented medium in the same order as in Figure 2 of the manuscript.

**Gene ID**	**Name**	***fold change***
ENSG00000130147	SH3BP4	1.401
ENSG00000172403	SYNPO2	1.403
ENSG00000089159	PXN	1.404
ENSG00000130347	RTN4IP1	1.404
ENSG00000108423	TUBD1	1.404
ENSG00000006576	PHTF2	1.408
ENSG00000100281	HMGXB4	1.408
ENSG00000173786	CNP	1.409
ENSG00000174177	CTU2	1.409
ENSG00000196498	NCOR2	1.409
ENSG00000104332	SFRP1	1.411
ENSG00000106628	POLD2	1.412
ENSG00000145386	CCNA2	1.416
ENSG00000104147	OIP5	1.417
ENSG00000086827	ZW10	1.421
ENSG00000126787	DLGAP5	1.421
ENSG00000111328	CDK2AP1	1.426
ENSG00000269958	AL049840.4	1.427
ENSG00000253954	HMGN1P38	1.429
ENSG00000203760	CENPW	1.430
ENSG00000138448	ITGAV	1.431
ENSG00000080839	RBL1	1.433
ENSG00000165490	DDIAS	1.435
ENSG00000100162	CENPM	1.439
ENSG00000115129	TP53I3	1.442
ENSG00000115325	DOK1	1.447
ENSG00000188229	TUBB4B	1.452
ENSG00000117593	DARS2	1.455
ENSG00000119326	CTNNAL1	1.457
ENSG00000060656	PTPRU	1.460
ENSG00000116791	CRYZ	1.461
ENSG00000159055	MIS18A	1.464
ENSG00000144354	CDCA7	1.464
ENSG00000107816	LZTS2	1.465
ENSG00000030110	BAK1	1.467
ENSG00000174021	GNG5	1.467

ENSG00000163629	PTPN13	1.468
ENSG00000117280	RAB29	1.470
ENSG00000173207	CKS1B	1.472
ENSG00000109390	NDUFC1	1.475
ENSG00000134569	LRP4	1.477
ENSG00000151503	NCAPD3	1.481
ENSG00000178999	AURKB	1.483
ENSG00000171241	SHCBP1	1.484
ENSG00000128228	SDF2L1	1.486
ENSG00000122756	CNTFR	1.495
ENSG00000102158	MAGT1	1.498
ENSG00000259917	HNRNPLP2	1.499
ENSG00000105968	H2AFV	1.500
ENSG00000153044	CENPH	1.511
ENSG00000106211	HSPB1	1.513
ENSG00000131873	CHSY1	1.521
ENSG00000105011	ASF1B	1.526
ENSG00000168078	PBK	1.530
ENSG00000197905	TEAD4	1.532
ENSG00000149929	HIRIP3	1.537
ENSG00000148219	ASTN2	1.561
ENSG00000149554	CHEK1	1.566
ENSG00000106105	GARS	1.566
ENSG00000187741	FANCA	1.568
ENSG00000090889	KIF4A	1.573
ENSG00000075188	NUP37	1.575
ENSG00000129173	E2F8	1.582
ENSG00000198860	TSEN15	1.582
ENSG00000254858	MPV17L2	1.585
ENSG00000066735	KIF26A	1.606
ENSG00000040275	SPDL1	1.612
ENSG00000072571	HMMR	1.619
ENSG00000125703	ATG4C	1.621
ENSG00000140545	MFGE8	1.636
ENSG00000136603	SKIL	1.637
ENSG00000149257	SERPINH1	1.646
ENSG00000165304	MELK	1.648
ENSG00000064042	LIMCH1	1.653
ENSG00000115163	CENPA	1.654
ENSG00000184260	HIST2H2AC	1.677
ENSG00000135862	LAMC1	1.689
ENSG00000162129	CLPB	1.689
ENSG00000173456	RNF26	1.699

ENSG00000183580	FBXL7	1.701
ENSG00000166002	SMCO4	1.704
ENSG00000072110	ACTN1	1.725
ENSG00000150961	SEC24D	1.727
ENSG00000125170	DOK4	1.730
ENSG00000140332	TLE3	1.736
ENSG00000268942	CKS1BP3	1.737
ENSG00000109790	KLHL5	1.743
ENSG00000114738	MAPKAPK3	1.746
ENSG00000164649	CDCA7L	1.751
ENSG00000129195	PIMREG	1.754
ENSG00000135736	CCDC102A	1.773
ENSG00000186350	RXRA	1.777
ENSG00000123473	STIL	1.786
ENSG00000185043	CIB1	1.800
ENSG00000144045	DQX1	1.811
ENSG00000168476	REEP4	1.844
ENSG00000153551	CMTM7	1.854
ENSG00000132423	COQ3	1.857
ENSG00000110108	TMEM109	1.873
ENSG00000163661	PTX3	1.961
ENSG00000175183	CSRP2	2.058
ENSG00000076356	PLXNA2	2.061
ENSG00000188486	H2AFX	2.075
ENSG00000095739	BAMBI	2.089
ENSG00000223764	AL645608.1	2.166
ENSG00000176014	TUBB6	2.195
ENSG00000179348	GATA2	2.251
ENSG00000115297	TLX2	2.255
ENSG00000101665	SMAD7	2.409
ENSG00000111145	ELK3	2.410
ENSG00000112559	MDFI	3.061
ENSG00000135111	TBX3	3.105
ENSG00000026025	VIM	3.167
ENSG00000107731	UNC5B	3.177
ENSG00000143320	CRABP2	3.328
ENSG00000168453	HR	3.855
ENSG00000064666	CNN2	6.016
ENSG00000115738	ID2	16.011
ENSG00000187634	SAMD11	24.625
ENSG00000125968	ID1	40.625

2 Supplementary Figures

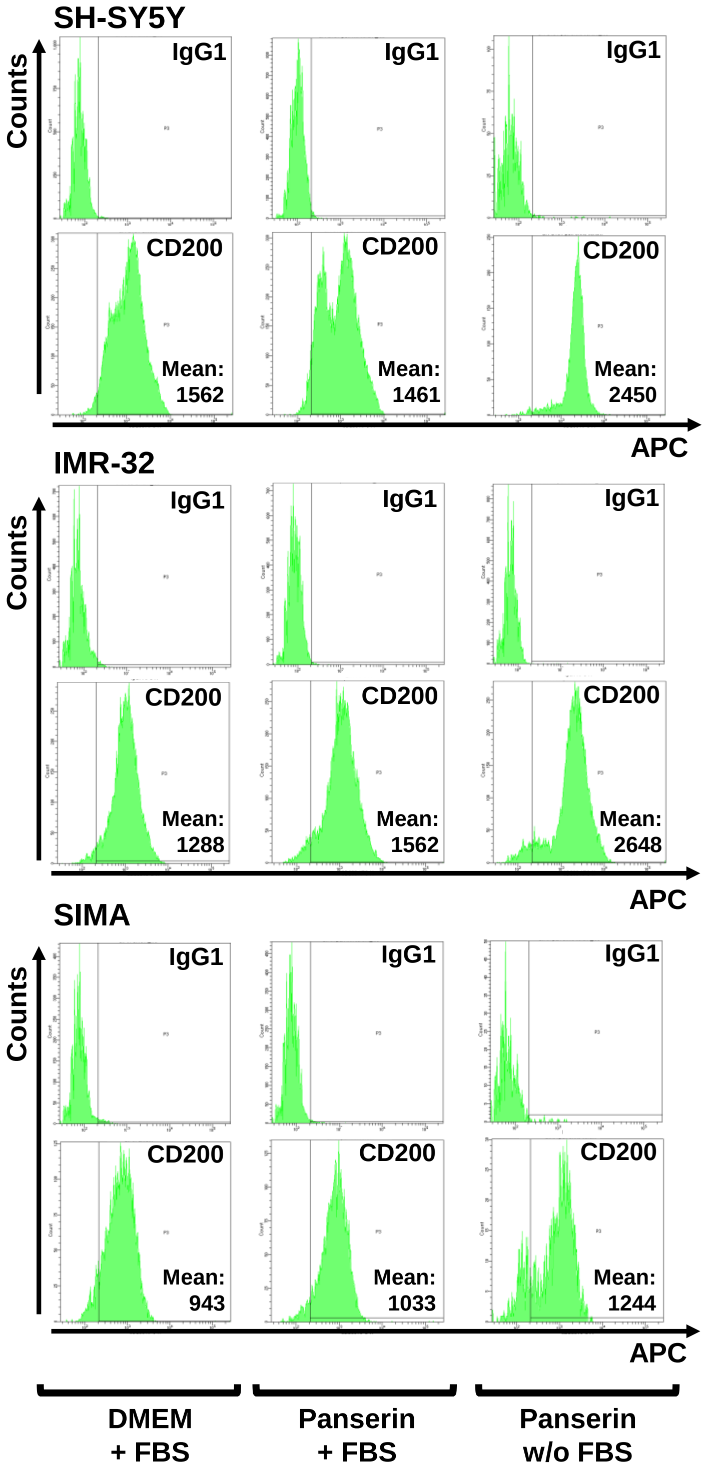

Supplementary Figure 1. Regulation of CD200 surface protein expression in three NB cell lines by medium-induced microenvironmental changes by flow cytometry. The histograms of NB cells stained with CD200-APC antibodies or isotype controls are shown. The mean fluorescence intensities of APC-positive cells are included.

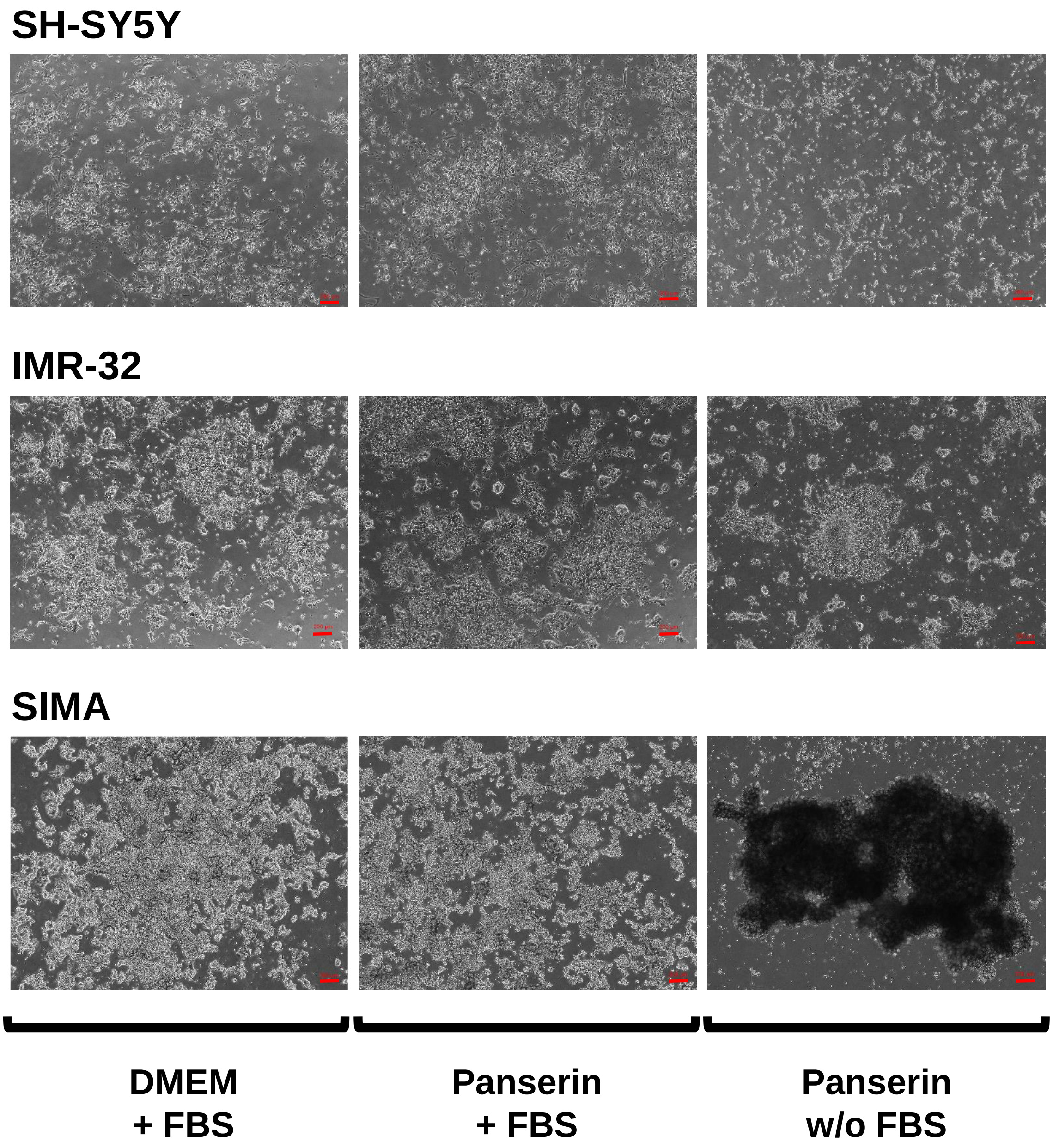

Supplementary Figure 2. Morphological analyses of three NB cell lines upon medium-induced microenvironmental change. For SiMa cells, a phenotype switching from loosely-adherent monolayers to low proliferating grape-like cellular aggregates was observed. Phase contrast microscopy on a Keyence microscope BZ-X810 was used. The bar represents 200 µm.
